# Ambient Ozone Concentrations Cause Increased Hospitalizations for Asthma in Children: An 18-Year Study in Southern California

**DOI:** 10.1289/ehp.10497

**Published:** 2008-03-06

**Authors:** Kelly Moore, Romain Neugebauer, Fred Lurmann, Jane Hall, Vic Brajer, Sianna Alcorn, Ira Tager

**Affiliations:** 1 Division of Biostatistics, School of Public Heath, University of California, Berkeley, California, USA; 2 Sonoma Technology, Inc., Petaluma, California, USA; 3 Department of Economics and Institute for Economic and Environmental Studies, California State University, Fullerton, California, USA; 4 Division of Epidemiology, School of Public Heath, University of California, Berkeley, California, USA

**Keywords:** air pollution, asthma, children, epidemiology, ozone

## Abstract

**Background:**

Asthma is the most important chronic disease of childhood. The U.S. Environmental Protection Agency has concluded that children with asthma continue to be susceptible to ozone-associated adverse effects on their disease.

**Objectives:**

This study was designed to evaluate time trends in associations between declining warm-season O_3_ concentrations and hospitalization for asthma in children.

**Methods:**

We undertook an ecologic study of hospital discharges for asthma during the high O_3_ seasons in California’s South Coast Air Basin (SoCAB) in children who ranged in age from birth to 19 years from 1983 to 2000. We used standard association and causal statistical analysis methods. Hospital discharge data were obtained from the State of California; air pollution data were obtained from the California Air Resources Board, and demographic data from the 1980, 1990, and 2000 U.S. Census. SoCAB was divided into 195 spatial grids, and quarterly average O_3_, sulfurdioxide, particulate matter with aerodynamic diameter ≤ 10 μm, nitrogen dioxide, and carbon monoxide were assigned to each unit for 3-month periods along with demographic variables.

**Results:**

O_3_ was the only pollutant associated with increased hospital admissions over the study period. Inclusion of a variety of demographic and weather variables accounted for all of the non-O_3_ temporal changes in hospitalizations. We found a time-independent, constant effect of ambient levels of O_3_ and quarterly hospital discharge rates for asthma. We estimate that the average effect of a 10-ppb mean increase in any given mean quarterly 1-hr maximum O_3_ over the 18-year median of 87.7 ppb was a 4.6% increase in the same quarterly outcome.

**Conclusions:**

Our data indicate that at current levels of O_3_ experienced in Southern California, O_3_ contributes to an increased risk of hospitalization for children with asthma.

In terms of numbers, morbidity burden, and health care costs, asthma is the most important chronic disease of childhood, with estimated medical care costs over $1 billion in 2005 (Wang et al. 2005). Based on its recent review of data on ozone-related health effects, the U.S. Environmental Protection Agency (EPA) has once again concluded that children with asthma constitute a group that is susceptible to O_3_- associated adverse effects on their disease (U.S. EPA 2006). Hospitalization and visits to emergency departments are major contributors to childhood asthma-related health care costs and account for approximately 12% of care costs for asthma in children 5–17 years of age (Wang et al. 2005). Despite the large number of studies on various asthma-related outcomes (symptoms, lung function) in relation to ambient O_3_, there are relatively few studies on O_3_-related hospital discharges and emergency department (ED) visits in children with asthma; and the results of these studies have not been consistent [U.S. EPA 2006 (Figures 7–8, 7–9)]. Moreover, these studies have been concerned with associations between pollutant exposures over a few days before hospital admission and over relatively short periods of calendar time.

Several studies illustrate findings based on short lag periods. White and colleagues (1994) reported that ED visits for asthma (1–16 years of age) to an Atlanta, Georgia, hospital increased by 37% on the 6 days in the summer of 1990 when the maximum 1-hr O_3_ concentrations exceeded 110 ppb. A subsequent Atlanta-based ecologic study reported that Medicaid claims for hospital admissions for asthma decreased during the time of the 1996 Summer Olympic Games in parallel with reductions of ambient O_3_ concentrations (Friedman et al. 2001). The decline in O_3_ was attributed to the marked decline in city traffic during the games, but associations with other mobile source emissions were not evaluated in the regression models. A third study from Atlanta for the summers of 1993–1995 found similar associations with ED visits, but these investigators could not separate effects due to particulate matter with aerodynamic diameter ≤ 10 μm (PM_10_) (Tolbert et al. 2000). An approximate 33% increase in ED visits for childhood asthma was reported from eastern Canada on days when the 1-hr maximum exceeded 75 ppb over the years 1984–1992, an association that was independent of concentrations of sulfate and total suspended particulates (TSP) (Stieb et al. 1996). Data from Washington, DC; Mexico City, Mexico; and Madrid, Spain, support these findings of O_3_-associated increases in ED visits, independent of pollens and PM_10_ (Babin et al. 2007; Galan et al. 2003; Romieu et al. 1995).

In contrast to the above results, several relatively recent European studies have not found these associations. Data from the APHEA (Air Pollution and Health: A European Approach) study from the period 1986–1992 from Barcelona, Spain; Helsinki, Finland; Paris, France; and London, UK, failed to find any association between ED visits and ambient O_3_ in children < 15 years of age (Sunyer et al. 1997). However, these results were based on O_3_ concentrations throughout all months of the year. Similarly, a study in London, based on year-long data for 12 EDs over the years 1992–1994, also failed to find any association between ED visits and hospitalizations for asthma and ambient O_3_ concentrations for children from birth to 14 years of age (Atkinson et al. 1999a, 1999b).

California’s South Coast Air Basin (SoCAB) has some of the highest concentrations of O_3_ in the U.S. [South Coast Air Quality Management District (SCAQMD) 2006] and will continue to be a major area of noncompliance under proposed new O_3_ standards (U.S. EPA 2005). Mobile source emissions are the main source of precursors for O_3_ generation (Fujita et al. 1992). Because O_3_ and other pollutant levels, in general, have been declining over the past 25 years (SCAQMD 2003), this area offers an excellent opportunity to study the relation between warm-season ambient O_3_ concentrations and hospitalizations for asthma in a large population that spans urban and rural areas. Therefore, we undertook an ecologic study of hospital discharges for asthma in children from 0 (birth) to 19 years of age over the period 1983–2000 in the SoCAB to evaluate the effect of population-level O_3_ exposure on asthma-related hospital discharge over time. Our approach is based on conventional linear modeling with adjustment for temporal factors that could confound the causal effect of interest. Our approach has several novel features: *a*) We used a very flexible, data-adaptive model fitting program that is based on multiple cross-validations (van der Laan and Dudoit 2003); *b*) pollutants other than O_3_ could enter our modeling at equivalent levels of complexity, as for O_3;_ and *c*) we used marginal structural models (MSM) to support the interpretation of population-level effects of O_3_ on the outcomes (van der Laan and Robins 2002).

## Methods

### Study area

**Figure 1:**
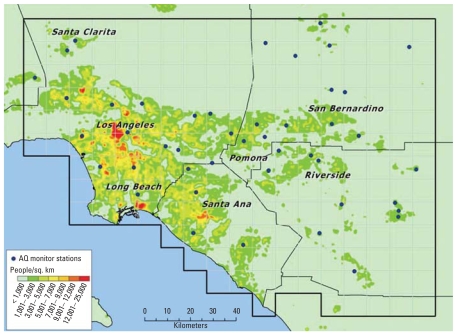
Study domain grid system with location of pollutant monitors and population density. AQ, air quality.

The study area was the portion of California’s SoCAB covered by the grids shown in Figure 1. The 20,000-km^2^ area extends from 34.6° latitude at its most northern reach to 33.2° latitude at its most southern extent. It is bounded on the west by −118.9° longitude and the Pacific Ocean, and extends to −116.8° longitude at its eastern end. We selected this location because it contained many areas that consistently exceeded National Ambient Air Quality Standards for O_3_ during the 1980–2000 study period (U.S. EPA 2000). Nonetheless, the area also experienced marked reductions in 1-hr and 8-hr maximum O_3_ concentrations over this time.

### Ambient pollutant data and exposure methods

We estimated the population’s exposure to O_3_, nitrogen dioxide, sulfur dioxide, carbon monoxide, PM with aerodynamic diameter ≤ 2.5 μm (PM_2.5_), and PM_10_ from ambient air quality measurements obtained from a network of stations that began monitoring for most of the pollutants before 1980. The number and locations of air monitoring stations (Figure 1) varied over the study period. The number of stations with valid air quality data in or near the grid in a given year varied from 45 to 55 for O_3_, 33 to 41 for NO_2_, 28 to 39 for CO, and 9 to 56 for PM_10_. We compiled quarterly average concentrations of the 1-hr daily maximum O_3_ and 24-hr average NO_2_, SO_2_, and CO from hourly measurements of gases. We compiled quarterly average concentrations of the 24-hr average PM_10_ and PM_2.5_ from monthly averages of every sixth day PM_10_ measurements and daily, every third day, and 2-week average PM_2.5_ measurements (Blanchard and Tanenbaum 2005). The air quality data were complemented with quarterly average daily 1-hr minimum and 24-hr average temperature and relative humidity data obtained from the SoCAB and National Weather Service measurements (National Climatic Data Center, NOAA Satellite and Information Service: http://www.ncdc.noaa.gov/oa/ncdc.html).

California PM_10_ data are not widely available before 1988. Special study PM_10_ mass data available in Burbank, downtown Los Angeles, Long Beach, Los Alamitos, Costa Mesa, Azusa, Rubidoux, Perris, and Banning were used for 1985–1987 (Solomon et al. 1988). Collocated PM_10_ and TSP data for 1988 through 1992 in Los Angeles, Orange, San Bernardino, and Riverside Counties indicated that daily PM_10_ concentrations were correlated with daily TSP and, on average, were 54% of TSP concentrations. The PM_10_ concentrations for 1980–1984 were estimated from the TSP data based on this relation. PM_2.5_ data were available for only 1994–2000.

The study domain was divided into two-hundred 10 km × 10 km spatial grids that covered the populated portion of the SoCAB, of which 195 were used. The population, other demographic, and health outcome data were aggregated into the grid cells [Supplemental Material, Figure S1 (online at http://www.ehponline.org/members/2008/10497/suppl.pdf)]. The air quality and meteorologic data were interpolated spatially from the monitoring stations to the grid cell centroids based on inverse distance-squared weighting. Maximum interpolation radii of 50 and 100 km were used for pollutants and meteorologic parameters, respectively. Although 100% of the grids had an O_3_ air quality station within 50 km, 73% of the grids had a station within 5–25 km of the grid centroids and 13% of grids had a station located within the grid on average [see Supplemental Material (online at http://www.ehponline.org/members/2008/10497/suppl.pdf) for other pollutant interpolation distances]. This interpolation approach worked reasonably well in this application, because the spatial coverage in the SOCAB monitoring network is good (typically, stations located 20–30 km apart); and spatial gradients in monthly average concentrations are modest.

Our principal exposure of interest was 1-hr daily, maximum O_3_. We chose this measure, because the same 1-hr maximum standard was in place for most of the study period; and the 1-hr maximum is the most commonly used metric in O_3_ epidemiologic studies. Quarterly, average O_3_ concentrations were low and showed little variability from October through March [see Supplemental Material, Figure S6 (online at http://www.ehponline.org/members/2008/10497/suppl.pdf) for sample quarters]. Therefore, we confined our analyses to April–June (quarter 2) and July–September (quarter 3), which constitute all months with the highest and most variable O_3_ concentrations.

### Hospital discharge and demographic data

Since 1983, hospital discharges (diagnoses, demographic data and medical payments) have been reported semiannually by all hospitals licensed in California. Patient-level data were extracted from a CD-ROM (Healthcare Information Resource Center, Sacramento, CA) and included: patient age category, county of residence and 5-digit ZIP Code, ethnicity, sex, major diagnostic category (plus four secondary), major procedure (plus four secondary), quarter admitted, length of stay, and hospital ID number (Office of Statewide Health Planning and Development, data files e-mailed July 2003). We focused on quarterly hospital discharges for asthma [*International Classification of Diseases, 9th Revision* (ICD-9; World Health Organization 1975) code 493, ICD-10 (World Health Organization 1993) code J45/46] listed as the first discharge diagnosis for children and adolescents from birth through 19 years of age. We included discharges in which the first listed diagnoses were acute sinusitis (ICD-9 461; ICD-10 J01) or pneumonia (ICD-9 480–483, 485–487; ICD-10 J10–J18) and asthma was the second listed diagnosis, because we could not be sure of the extent to which the presence of asthma actually led to the hospitalization [see Supplemental Material (online at http://www.ehponline.org/members/2008/10497/suppl.pdf)].

We obtained data from the U.S. Census Bureau’s decadal surveys for years 1980, 1990, and 2000 [see Supplemental Material (online at http://www.ehponline.org/members/2008/10497/suppl.pdf)]. We reviewed all income, demographic, and residential data and selected covariates that were considered likely to affect asthma morbidity and were likely to show spatial clustering and temporo-spatial trends (graphs available on request from authors). We selected 57 sociodemographic variables.

The finest spatial resolution for which hospital discharge data were available was the 5-digit postal ZIP code of the patient’s residence; the patient’s street address, 9-digit ZIP code, or census block were not available. Population-weighted ZIP-to-grid allocation factors were developed with geographic information system (GIS) tools for 1980–1984, 1995–1994, and 1995–2000. Separate allocations factors were developed for males and females for < 1 year and 1–19 years of age. [see Supplemental Material for details (online at http://www.ehponline.org/members/2008/10497/suppl.pdf)].

Spatial allocation of demographic data to exposure grids was based on the smallest geographic unit for which census data were available. We used GIS software (ArcGIS9; ESRI, Redlands, CA) to map the demographic data to grids. Eight population variables from 1980 and one population variable from 1990 and 2000 were renormalized after the spatial allocation to insure consistency across census topics (e.g., population by race was normalized by the total population; population by sex, age, and race was normalized for consistency with population by race and population by sex). Population and other demographic parameters were estimated for the intracensus years by linear interpolation of the gridded data for 1980, 1990, and 2000.

### Data analysis.

#### Data structure

The data consist of 195 geographic units (grids) with quarterly measurements from 1983 through 2001 that include 14,040 records and 72 quarters for children birth to 19 years of age. We calculated the proportion of asthma-related hospital discharges as the number of asthma-related hospital discharges in each grid in each quarter divided by the total population birth to 19 years of age in the corresponding grid and quarter. After removal of nine outliers, we used data for quarters 2 and 3 only (7,011 observations).

There were no missing values for the proportion of asthma-related discharges or quarterly O_3_. Among the 47 covariates considered, 35 had no missing values. Among the 12 remaining covariates, the proportion of missing values ranged from 0.4% to 6.2%.

#### Statistical models

We denote the observed data structure by *O* = [*W̄*(71),*Ā*(71,*Ȳ*(72)] representing quarterly measurements from time 0 to 72 of the confounders, O_3_ levels and proportion of asthma-related hospital discharges: *a*) The history of O_3_ is denoted by *Ā*(71) = [*A*(0), …, *A*(71)], and *A*(*t*) represents the O_3_ level measured at time *t; b*) the history of asthma-related hospital discharges as a percentage of the total area-specific population is denoted by *Ȳ*(72) = [*Y*(1),…,*Y*(72)], and *Y* (*t*) represents the proportion of asthma discharges measured at time *t*; and *c*) the history of potential time-dependent confounders of the effect of O_3_ on asthma-related hospital discharges is denoted by *W̄*(*K* ) = [*W*(0), …,*W*(*K* )], where *W*(*t*) is a multivariate vector of potential confounders measured at time *t*: socioeconomic and demographic variables, co-pollutants, and meteorologic variables.

Our modeling approach aims at the investigation of the effect of *A*(*t* – 1) on *Y*(*t*). In this study, the outcome at time *t* [*Y*(*t*)] and exposure at time *t* – 1 [*A*(*t* – 1)] are actually measured during the same quarter, which does not violate the time-ordering assumption on which are based valid causal inferences (the exposure precedes the outcome). Because we consider the effect of O_3_ on asthma-related hospital discharges collected during quarters 2 and 3 only, we thus have 36 outcomes of interest rather than 72.

A typical assumption that is often not stated explicitly is that the observed data consist of *n* independent and identically distributed observations from the random variable *O* with distribution *P*. In this analysis, we make the assumption that the observed data consist of *n* = 195 random variables *O_i_* that describe each spatial/geographic unit *i*, *i* = 1,…, *n*, each with distribution *P_i_*. Under this assumption, it follows that mutual independence between the random variables *O_i_*, conditional on the exposure regimen, is a reasonable approximation [see Supplemental Material for additional details (online at http://www.ehponline.org/members/2008/10497/suppl.pdf)].

We chose to investigate the effect of O_3_ on the asthma-related hospital discharge proportion for quarterly exposure to O_3_ only; that is, we did not consider the effect of an O_3_ history over multiple quarters. This decision was motivated by our view that most of the effect of O_3_ could be captured by the exposure period of only one quarter—by estimation of the effect of O_3_ during a given quarter on the outcome during that same quarter in the seasons with the highest levels of O_3_. Because the experimental units are geographic areas rather than individuals, the population in the units was constantly changing over the 18-year study period; however, within a given quarter, the population was relatively stable. Another reason for selection of the short exposure period relates to power (sample size, *n* = 195) for identification of effects that extend over a longer exposure period (Neugebauer et al. 2007).

We estimated this effect of O_3_ on the proportion of asthma-related hospital discharges with two approaches: the traditional method of regression of the proportion of asthma-related hospital discharges on O_3_ and confounder; and a method based on history-restricted marginal structural models (HRMSMs) (Neugebauer et al. 2007). In contrast to the usual MSM approach, HRMSMs allow the investigator to specify the time interval over which the history of exposure is to be considered—a critical issue for this analysis.

For both approaches, working models considered were semiparametric linear models. The rationale for use of linear models is presented in the Supplemental Material (online at http://www.ehponline.org/members/2008/10497/suppl.pdf).

The deletion/substitution/addition (DSA) algorithm was used for all model selections required for the traditional approach and the nuisance parameters in the HRMSM approach (Sinisi and van der Laan 2004). This is a data-adaptive model selection procedure based on cross-validation that relies on deletion, substitution, and addition moves to search through a large space of possible polynomial models. The criterion for model selection is based not on *p*-values but on a loss function (empirical and cross-validated residual sum of squares). The DSA procedure is publicly available as an R package (http://www.stat.berkeley.edu/~laan/Software/). All 7,011 observations were provided to all DSA runs. The DSA assumes that data are missing at random when searching for the best predictive linear model of the proportion of asthma-related hospital discharges.

#### Traditional regression approach

The traditional approach to estimate the effect of *A*(*t* – 1) on *Y* (*t*) is to regress the outcome, *Y*(*t*), on the exposure, *A*(*t* – 1), and all confounders. Potential confounders are: *W̄*(*t* – 1) = [*W*(1), …,*W*(*t* – 1)]), *Ȳ*(*t* – 1), and *Ā*(*t* – 1). Under the assumption of no unobserved confounders, this approach allows the investigation of the effects of O_3_ at each quarter, *A*(*t* – 1), on *Y*(*t*), conditional on the past confounders in the regression model. It is realistic to assume that O_3_ levels before quarter *t* [i.e., *Ā*(*t* – 1)] do not affect the outcome in quarter *t*; thus, we did not consider them as confounders. Similarly, we did not consider past quarter discharges [i.e., *Ȳ*(*t* – 1)]. Among all potential covariates *W̄*(*t* – 1), we only considered as potential confounders all same-quarter covariates *W*(*t* – *1*) and only copollutants and meteorologic variables from the previous quarter and previous year included in *W*(*t* – 2) and *W*(*t* – 5). This allowed us to maximize control of possible long-term trends in other pollutants on the current quarter’s outcome. Forty-seven remaining covariates were identified as potential confounders of the effect of O_3_ at quarter *t* – 1 on the proportion of asthma-related hospital discharges at quarter *t*. Among these 47 covariates, only 29 were considered in the analysis, based on their univariate association with the proportion of asthma-related hospital discharges and O_3_ levels [see Supplemental Material, Table S1 (online at http://www.ehponline.org/members/2008/10497/suppl.pdf)]; this subset of 29 potential confounders is denoted with *W̄* *×* (*t* – 1).

We selected a pooled model for *E*[*Y*(*t*)| *A*(*t* – 1),*W̄* *×* (*t* – 1)] across time with the DSA [see Supplemental Material (online at http://www.ehponline.org/members/2008/10497/suppl.pdf)]. The standard errors for the coefficients in the selected model were obtained with the generalized estimation equation procedure (semiparametric modeling with the independence correlation structure).

This traditional approach does not answer directly our original question of interest: the population-level effect of *A*(*t* – 1) on *Y*(*t*); indeed, this method provides the estimate of the effect conditional on confounders *W̄* *×* (*t* – 1) which only correspond with the population-level effect estimate of interest when confounders are not effect modifiers.

#### HRMSM

To obtain an estimate of the population-level, causal effect of O_3_ on the proportion of asthma-related hospital discharges, we applied an HRMSM (Neugebauer et al. 2007). HRMSMs have been developed to address situations where only part of the exposure history is relevant [for details, see Supplemental Material (online at http://www.ehponline.org/members/2008/10497/suppl.pdf)]. The exposure period considered is a single quarter as opposed to the entire exposure history.

We implemented two estimators of HRMSM causal parameters: the inverse probability of treatment weighted (IPTW) and G-computation. Confidence intervals (CIs) and *p*-values for the two estimates were obtained with 10,000 bootstrap iterations, where resampling was based on the 195 independent grids.

## Results

Characteristics of the total population who resided in the study domain (Figure 1) over the 84 quarters (1980–2000) are summarized in Table 1. For a summary of the characteristics of the population of asthma discharges from birth to 19 years of age for quarters 2 and 3 for 1983–2000, see Supplemental Material, Table S4 (online at http://www.ehponline.org/members/2008/10497/suppl.pdf).

**Figure 2:**
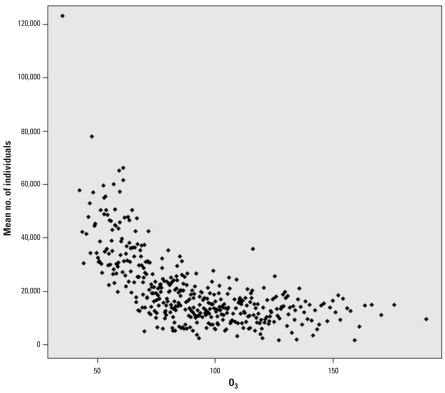
Distribution of population number by 400 quantiles of quarterly 1-hr maximum O_3_ over quarters 2 and 3, 1983–2000.

O_3_ concentrations declined steadily over the entire study period [Supplemental Material, Figure S8_a (online at http://www.ehponline.org/members/2008/10497/suppl.pdf)]. Median 1-hr maximum and 8-hr average median O_3_ for quarters 2–3 declined across all grids (Figure 2). Median 1-hr maxima also declined in quarters 1 and 4 [Supplemental Material, Table S5 (online at http://www.ehponline.org/members/2008/10497/suppl.pdf)]. Substantial declines were seen for the other pollutants as well [see Supplemental Material, Table S5, Figure S8_b (online at http://www.ehponline.org/members/2008/10497/suppl.pdf)]. The distribution of the quarterly population was skewed toward areas at the lower two-thirds of the quarterly O_3_ distributions [see Supplemental Material, Figure S9 (online at http://www.ehponline.org/members/2008/10497/suppl.pdf)]. During 1980–2000, 25.7% of the gridded, quarterly, average 1-hr maximum O_3_ concentrations exceeded the level of California’s daily 1-hr standard (90 ppb), and 8.2% exceeded the federal daily 1-hr standard of 0.12 ppm. For quarters 2 and 3 and years 1983 through 2000, 47.5% and 13.2% of the quarterly, average, 1-hr maximum O_3_ concentrations exceeded the California and federal daily 1-hr standard, respectively (California EPA 2008; U.S. EPA 2000).

The median 1-hr and 8-hr maximum average O_3_ levels were highly correlated (*r* = 0.99) (Table 2). During 1980–2000, O_3_ concentrations showed moderate correlation with PM_10_ and little correlation with the other pollutants. O_3_ and PM_10_ are correlated on a quarterly averaging time, because wind-blown dust and resuspended road-dust emissions cause relatively high PM_10_ levels during the dry season when O_3_ levels also are high.

In the conventional regression model, the identical model was selected when O_3_ was forced into the model or when the DSA was free to choose any variable (Table 3). Of the seven [of 29; see Supplemental Material, Table S1 (online at http://www.ehponline.org/members/2008/10497/suppl.pdf)] other variables selected into the models, none was another pollutant (Table 3) [see Supplemental Material for details of model selection (online at http://www.ehponline.org/members/2008/10497/suppl.pdf)]. Thus, it is unlikely that the association is confounded by other pollutants. In addition, time was not selected as a main effect or interaction variable—an observation indicating that the unit effect of O_3_ on the proportion of asthma-related discharges was constant over the study period, despite the decline in the levels of O_3_ and all other pollutants measured. The estimated effect of a 10-ppb increase in the quarterly average 1-hr maximum O_3_ was 1.4 discharges per 105 age-eligible population (95% CI, 0.71–2.09 per 105 population). The final model was used to predict the proportion of discharges at the median O3 concentration (87.7 ppb) over all grids and all quarters (3.12 × 10–4). A 10-ppb increase above this level is estimated to lead to a 4.6% increase in the proportion of discharges (3.26 × 10–4).

**Figure 3:**
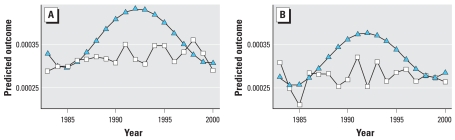
Predicted proportions of quarterly hospital discharges based on a model that included only time variables (triangles) and the model in Table 4 that includes O_3_ and the demographic variables (squares) for quarters 2 (*A*) and 3 (*B*).

To determine the extent to which time contributed to confounding, the DSA was run first only with time variables. When the time variables selected by the DSA were forced into a model that also forced in O_3_ into the same model, no other variables were selected by the DSA. This indicates that the demographic variables included in the models in Table 3 were capturing the overall temporal confounding related to population demographic and other unmeasured time-varying factors. This is seen clearly in Figure 3. The model with only time variables shows a clear temporal trend in hospital discharges. In contrast, the model with O_3_ and demographic variables shows a nearly constant proportion of hospital discharges over the study quarters.

To provide population-level estimates of pollutant effects, we used G-computation and IPTW to fit an HRMSM. Treatment models (models that relate cofounders to quarterly O_3_ concentrations and include other confounding variables) on which IPTW estimation relies [Supplemental Material (online at http://www.ehponline.org/members/2008/10497/suppl.pdf)] demonstrated that the experimental treatment assignment assumption was not tenable. We applied a diagnostic tool to assess the bias in the IPTW estimator due to the experimental treatment assignment (ETA) violation (Wang et al. 2006) and showed a 76% bias in comparison to the G-computation estimate (Table 4). Therefore, we relied on the G-computation estimator. The interpretation of the MSM parameter estimate is as follows: If, contrary to fact, the population experienced a 10-ppb increase in quarterly O_3_, then hospital admissions would increase by 1.4 × 10^5^ age-eligible population at any given quarter. This would represent the same effect estimated by the conventional regression analysis. In other words, the results from the MSM and conventional analyses, in this particular analysis, give identical parameter estimates because there are no interaction terms in the conventional model.

## Discussion

The most recent U.S. EPA synthesis of ambient O_3_ health effects concludes that children with asthma suffer acute adverse health consequences at current ambient levels of O_3_ (U.S. EPA 2006). Among these adverse outcomes, asthma-related hospital discharges are based on some of the least consistent data (see Figure 7–9, U.S. EPA 2006). In some studies, asthma discharges are not separated from other respiratory diseases of childhood (e.g., Burnett et al. 2001). Although some of the inconsistency likely relates to differences in populations and pollutant mixtures, some of it also could relate to the relatively short time periods (Atkinson et al. 1999a, 1999b) and special circumstances (Friedman et al. 2001) under which the data were collected and the inability to separate O_3_ effects from those of other pollutants (Tolbert et al. 2000) The present ecologic study addresses these problems through evaluation of the relation between hospital discharges for asthma for infants, children, and adolescents and changes in warm-season ambient O_3_ concentrations in a large, ethnically/racially diverse region of Southern California over 18 years (1983–2000). This region has seen changing pollutant levels and population structure over both time and space during the study period. Of note is the decline in the percentage of native-born residents, from 40% in 1980 to 30% in 2000, and the decline in the percentage who listed their primary race/ethnicity as Caucasian, from approximately 80% in 1980 to approximately 60% in 2000 [see Supplemental Material for additional details (online at http://www.ehponline.org/members/2008/10497/suppl.pdf)].

Our data indicate that, despite consistent and substantial declines in ambient warm-season O_3_ concentrations in the area of study (Figure 3A), there has been a time-independent, constant effect of ambient levels of O_3_ on quarterly hospital discharge rates for asthma. For example, we estimate that the average effect of a 10-ppb mean increase in mean quarterly 1-hr maximum O_3_ over the 18-year median of 87.7 ppb was a 4.6% increase (point estimate) in quarterly hospital discharges for asthma (increase from 3.12 to 3.26 × 10^−4^ age-eligible population) over a time period in which the median age-eligible population was approximately 4 million persons. Moreover, from a regulatory policy perspective, if the national 8-hr maximum were set at 75 ppb instead of 70 ppb (~ 6.6 ppb difference in the 1-hr max), our results suggest that there could be an excess of O_3_-season, asthma-related hospital admissions for children in the study area of approximately 3.0% (point estimate) above what could be expected at a more protective standard. Further, our data indicate that the O_3_-related asthma discharges (the pollutant mixture remaining the same) would be affected by changes in demography that likely will occur; and caution needs to be exercised in terms of extrapolation into the future.

Several features of our analysis strengthen the quantitative estimates and the apparent lack of time dependence of the O_3_ effect:

First, we used a very flexible, multiple cross-validation model fitting algorithm in which the constraints on the model were as follows: *a*) maximum model size of 10 variables; *b*) maximum power of any individual variable (includes time) of 3; and *c*) a maximum of two-way interactions between the 29 covariates considered, such that the sum of powers of each covariate in the interaction term is ≤ 3. Thus, the flexibility of the models allowed the description of complex associations between changing demography, meteorologic conditions, and all temporal confounders for which time was a surrogate. A direct by-product of this flexible model fitting is that the form of the O_3_–hospital discharge relation was free to take any polynomial form over time. This approach is similar in flexibility to model fitting with spline functions.

Second, the 24-hr concentrations of PM_10_, NO_2_, and CO could enter the model at equivalent levels of complexity as O_3_ and any other covariate and in interaction with time. Thus, we did not start with the *a priori* assumption that warm-season O_3_ would be the only or the most important component of the four pollutants for which we warm-season data.

Third, we ran our analysis 10 separate times, each time with a different split for cross validation (equivalent to 50 splits of the data). All model runs selected O_3_ and no other pollutant, and the identical model with covariates was selected 8 of 10 times (Table 3).

Fourth, we used an MSM approach to investigate the marginal (population-level) effects of O_3_ on the outcome (Robins et al. 2000). This approach approximates what would have been observed if we could have randomized all of the spatial units at each time point to a quarterly mean O_3_ concentration. The results of this analysis indicated that the conventional statistical association model, in this particular analysis, was equivalent to the G-computation estimates of the HRMSM parameters—an observation that is not surprising, given that there were no interactions in the association model. Therefore, under certain assumptions noted above, the O_3_ parameter (Table 3) can be interpreted as a causal, unconditional (i.e., not stratum specific) population-level effect estimate. In other words, if, contrary to fact, the median quarterly average 1-hr maximum increased by 10 ppb in all geographic units, the quarterly average hospital discharge rate would be expected to increase by 1.4 discharges/10^5^ age-eligible population. This causal interpretation relies on the counterfactual framework embodied in HRMSMs (Neugebauer et al. 2007), particularly the assumption of no unmeasured confounders. The fact that, in our analyses, the association between O_3_ hospital discharges can be interpreted further as the population-level effect estimate of O_3_ based on the G-computation estimator of an HRMSM relies on the critical assumption that the conventional associational model selected with the DSA algorithm is correctly specified (particularly the absence of interaction terms between O_3_ and covariates), the assumption of correct model specification is embodied in all analyses of observational data. The inference for this causal effect estimate was obtained by bootstrap (without consideration of additional variability introduced by the model selection procedure as is the case with virtually all reports of conventional analyses). We are exploring alternate causal estimators of causal parameters that do not rely on the ETA assumption to validate the results presented in this paper to further verify the validity of the inference, and a preliminary assessment of this latter analysis is supportive. The full results of this alternative analysis are the subject of a subsequent paper.

Finally, the results demonstrate that the addition of O_3_ and demographic variables to our analyses removed all of the time trend in the hospital discharge data (Figure 3). Finally, although we included discharges with a primary diagnosis of pneumonia or acute sinusitis, we do not think that this has biased our results. Our estimate of the median quarterly discharge rate for asthma is at the lower end of such estimates for all or part of the age range that we included (National Center for Health Statistics 2004).

It is difficult to compare our results with other studies because we used a different time reference—3-month intervals—in contrast to a daily time metric in most other studies (Burnett et al. 2001; Friedman et al. 2001). The most important factor that governed the choice of the time metric related to the fact that, for practical purposes, O_3_ is an outdoor pollutant whose indoor concentrations are determined by household ventilation (open windows, use of air conditioners) (Gonzales et al. 2003). Because people of all ages spend most of their days indoors (Wiley et al. 1991a, 1991b), we reasoned that a 3-month interval, based on typical patterns of O_3_ concentrations to which people would be exposed during their times out of doors, would provide a more stable population-level estimate than would be the case for shorter time intervals, such as days or weeks. Several consequences stemmed from this choice. Because most studies of the health effects of short-term exposures to O_3_ indicate that O_3_ impacts on health occur within a few days after exposures (Galan et al. 2003; Mortimer et al. 2002), we did not feel that it was justified to lag population exposure by 3 months (i.e., one quarter). Therefore, we related O_3_ concentration in a given quarter to hospital discharges in that quarter. On its face, this would appear to violate the requirement for preservation of temporal sequence. However, given that we used average hospital discharges at the end of a quarter, this choice is valid. Furthermore, each of the 195 spatial units was assigned its spatially specific average quarterly discharge rate and O_3_ concentration, and the data were treated as a repeated-measures problem over the 36 quarters; we have accounted for differences in the mean exposure over space and time. In this regard, some daily time-series studies may have violated the temporality assumption in that their designation of lag 0 often includes the day of hospital admission. To be sure that longer-term trends for other pollutants did not confound our O_3_ exposure estimates, we considered previous quarter and previous year PM_10_, NO_2_, and CO.

The potential for spatial correlation to result in incorrect variance estimates for exposure outcome measures in time series studies of health effects of air pollutants has been noted (Ramsay et al. 2003a, 2003b). Although we did not perform a time-series analysis, we did address the issue of spatial correlation by not assuming that the data for each unit are obtained from independent draws from a common distribution but rather from each of 195 distributions whose similarity can be explained by close geographic proximity, conditional on the exposure regimen, and thus the independence assumption is reasonable.

Although we report the results as “O_3_-related effects,” O_3_ is likely to be the best marker (of the pollutants available for analysis) for the gaseous oxidant species produced by the complex photochemistry that occurs in the SoCAB during the warm months of the year and involves oxides of nitrogen and hydrocarbons, largely from mobile source emissions. O_3_ is the most abundant oxidant in the urban atmosphere; however, the mixture also includes peroxyacetylnitrate, hydrogen peroxide, organic peroxides, and the hydroxyl, hydroperoxy, and many organic peroxy radicals (Atkinson 1997). Several epidemiologic studies have shown that the oxidant properties of ambient air contribute to adverse health outcomes in persons with and without asthma (Grievink et al. 1998; Romieu et al. 1996, 1998, 2002). For example, Romieu et al. (2002) studied asthmatics in Mexico City and demonstrated that among the pollutant measurements for SO_2_, PM_10_, NO_2_, and O_3_, O_3_ was most closely associated with decrements in lung function in children and were reversed by antioxidant vitamin supplementation. Relevant to our study, the effects were most marked in those with severe asthma—the pool of subjects out of which hospital admissions are most likely to occur. The findings in these studies have been supported by controlled O_3_ exposure studies in which subjects were placed on diets supplemented with antioxidant vitamins and vegetable oils (Samet et al. 2001) and studies of airways reactivity after controlled O_3_ exposure (Trenga et al. 2001).

In summary, we conducted exhaustive analyses to address many of the outstanding issues related to reported associations between O_3_ and use of hospital services for asthma. Although additional work is ongoing to buttress the causal interpretation that we have given to our results, our data support and extend other observations that ambient O_3_ (highly oxidant, ambient, warm-season environments) causes increases in hospital admissions in children with asthma. Moreover, the linearity of the relation that we observed indicates that these excess asthma hospital discharges can be expected to continue at levels of air quality experienced in southern California.

## Figures and Tables

**Table 1:** Selected demographic variables, all ages: 1980–2000.

Variable	Spatial grid values for 84 quarters [median (IQR); range]
Total population (no.)	13,209,192 (11,847,989–14,047,041; 10,572,161–14,785,147)
Race (%)	
Hispanic	14.8 (9.2–23.7; 0–78.8)
Caucasian	75.9 (61.8–84.4; 1.9–100)
African American	2.1 (0.9–4.9; 0–55.4)
Asian	2.9 (1–6.2; 0–31.6)
Other	1.5 (0.8–2.6; 0–71.7)
Residence (%)	
Same house entire period	43.3 (36.5–49.5; 0–100)
Different house, same county	30.2 (24.7–35; 0–61.2)
Different California county	15.1 (6.5–25.5; 0–100)
Different state	9.3 (6.8–12.1; 0–58.5)
Unemployed (all ages)	35.3 (30.5–42.6; 17.8–72.8)
Below poverty level	9.4 (6–13.1; 0–100)

IQR, interquartile range. Range is 25th–75th percentile.

**Table 2:** Correlations between pollutants for SoCAB, all quarters, 1980–2000.

	1-hr O_3_ max	8-hr O_3_	24-hr NO_2_	24-hr CO	24-hr SO_2_	24-hr PM_10_
1-hr O_3_ maximum	1	0.99	0.16	−0.12	−0.06	0.52
8-hr O_3_		1	0.05	−0.21	−0.14	0.46
24-hr NO_2_			1	0.76	0.51	0.53
24-hr CO				1	0.60	0.36
24-hr SO_2_					1	0.13
24-hr PM_10_						1

**Table 3:** Conventional regression analysis of association between quarterly 1-hr maximum O_3_ concentrations (ppb) and hospital discharges for asthma, birth to 19 years of age: SoCAB 1983–2000.

Regression^a^	O_3_ parameter estimate	Robust SE	*p-*Value
O_3_ forced into regression b	1.4 × 10^−6^	3.5 × 10^−7^	5.0 × 10^−5^
O_3_ not forced into regression	1.4 × 10^−6^	3.5 × 10^−7^	5.0 × 10^−5^

a The DSA was run with 29 candidate variables that were marginally associated with the outcome and O_3_ (p-value < 0.05). The DSA was run with 10 different random data splits and selected the same model for all 10 runs for the forced model and 8 times for the unforced model. The two models that differed included an O_3_ term. PM_10_ was not selected into the analyses when either all measurements were used or when measurements were restricted to those directly measured (1988–2000). To convert 1-hr maximum O_3_ to 8-hr (0100–1800 hours) mean, divide the 1-hr maximum by the following conversion factor: 1.3279 (± 9.97 × 10^−4^). For example, an 8-hr maximum of 70 ppb corresponds to a 1-hr maximum of 93 ppb in our data. The conversion factor is based on the linear regression of 1-hr maximum on the 8-hr maximum (t-value, 1330.58).

b The DSA selected 7 other terms in addition to O_3_: white race, white race^3^ (cubed), income = $20,000–$39,999, average temperature for the quarter, relative humidity^3^, median income as a continuous variable, foreign born. Units are increases in discharges in total age-specific population per ppb (see Supplemental Material for details).

**Table 4:** Marginal structural model analysis of causal association between quarterly 1-hr maximum O_3_ concentrations (ppb) and hospital discharges for asthma, birth to 19 years of age: SoCAB 1983–2000.

MSM estimator	Parameter estimate	SE	*p*-Value
G-computation	1.4 × 10^−6^	3.6 × 10^−7^	5.5 × 10^−5^
IPTW^a^	2.9 × 10^−7^	3.5 × 10^−7^	0.41
Bias due to ETA violation (%)^a^	76		

a ETA bias estimated using a diagnostic tool based on parametric bootstrap sampling from an estimated data-generating distribution to Wang et al. (2006).
